# Antioxidants in Age-Related Macular Degeneration: Lights and Shadows

**DOI:** 10.3390/antiox14020152

**Published:** 2025-01-27

**Authors:** Uday Pratap Singh Parmar, Pier Luigi Surico, Tommaso Mori, Rohan Bir Singh, Francesco Cutrupi, Pramila Premkishore, Gabriele Gallo Afflitto, Antonio Di Zazzo, Marco Coassin, Francesco Romano

**Affiliations:** 1 Department of Ophthalmology, Government Medical College and Hospital, Chandigarh 160047, India; 2 Department of Ophthalmology, Campus Bio-Medico University Hospital, 00128 Rome, Italy; 3 Department of Sense Organs, La Sapienza University, 00185 Rome, Italy; 4 Department of Health and Medical Sciences, Adelaide Medical School, Adelaide, SA 5000, Australia; 5 Department of Biochemistry and Molecular Biology, Augusta University, Augusta, GA 30912, USA; 6 Ophthalmology Unit, Department of Experimental Medicine, University of Rome “Tor Vergata”, 00128 Rome, Italy; 7 Eye Clinic, Department of Biomedical and Clinical Sciences, Ospedale Luigi Sacco, University of Milan, 20157 Milan, Italy

**Keywords:** age-related macular degeneration, AMD, antioxidants, oxidative stress

## Abstract

Age-related macular degeneration (AMD) is a leading cause of vision impairment worldwide, primarily driven by oxidative stress and inflammation. This review examines the role of antioxidants in mitigating oxidative damage, emphasizing both their therapeutic potential and limitations in AMD management. Key findings underscore the efficacy of specific antioxidants, including vitamins C and E, lutein, zeaxanthin, and Coenzyme Q10, in slowing AMD progression. Landmark studies such as AREDS and AREDS2 have shaped current antioxidant formulations, although challenges persist, including patient variability and long-term safety concerns. Emerging therapies, such as mitochondrial-targeted antioxidants and novel compounds like saffron and resveratrol, offer promising avenues for AMD treatment. Complementary lifestyle interventions, including antioxidant-rich diets and physical activity, further support holistic management approaches. This review highlights the critical role of antioxidants in AMD therapy, advocating for personalized strategies to optimize patient outcomes.

## 1. Introduction

Age-related macular degeneration (AMD) is a leading cause of vision impairment and blindness, particularly among older adults worldwide. It significantly affects quality of life by compromising central vision, thereby limiting activities such as reading, driving, and recognizing faces. AMD poses substantial challenges to personal independence and economic productivity.

AMD is broadly classified into two main forms: dry (non-exudative or atrophic) and wet (exudative or neovascular). Dry AMD is the most common type, accounting for approximately 85–90% of cases. It progresses through early, intermediate, and advanced stages, with advanced dry AMD leading to geographic atrophy (GA) [1]. GA is characterized by the progressive loss of retinal pigment epithelium (RPE) and photoreceptor cells, resulting in slow but severe central vision loss [2]. In contrast, wet AMD, though less prevalent, is responsible for the majority of severe vision loss. It involves abnormal macular neovascularization (MNV) that breaches Bruch’s membrane, causing fluid accumulation, hemorrhage, and scar formation that rapidly compromise vision [1].

Despite advancements in anti-VEGF therapies for wet AMD, treatment limitations persist, including the need for frequent intravitreal injections and incomplete response in some patients. For dry AMD, there are currently no approved treatments to halt its progression, highlighting a critical unmet medical need [3]. Addressing these challenges necessitates a better understanding of AMD pathogenesis, where oxidative stress plays a central role.

Oxidative damage, driven by an imbalance between reactive oxygen species (ROS) and antioxidant defenses, contributes significantly to AMD pathology. The retina’s high metabolic rate, constant exposure to light, and oxygen-rich environment make it highly susceptible to oxidative stress. The RPE and photoreceptor outer segments are particularly vulnerable, where accumulated oxidative damage leads to cellular dysfunction, drusen formation, and chronic inflammation.

This review explores the potential of antioxidants in AMD management. It evaluates both established and emerging therapies, considering their mechanisms of action, efficacy, and limitations. By highlighting key findings from landmark trials like AREDS and AREDS2, as well as novel strategies such as mitochondrial-targeted antioxidants and nanoceria particles, we aim to provide insights into optimizing therapeutic approaches to meet the pressing needs of AMD patients.

## 2. Materials and Methods

The review was conducted using two key resources: PubMed (https://pubmed.ncbi.nlm.nih.gov, (accessed on 12 January 2025)) and Reference Citation Analysis (RCA) (https://www.referencecitationanalysis.com, (accessed on 12 January 2025)). PubMed, a trusted and widely utilized biomedical literature database maintained by the National Library of Medicine (NLM), served as the primary platform for this research.

A systematic approach was employed, using a combination of search terms that included variations of “Antioxidants” and “Dietary Supplementation” in conjunction with terms related to “age-related macular degeneration (AMD)”. Boolean operators (AND, OR, NOT) were applied to logically structure the queries, ensuring comprehensive coverage of relevant studies while filtering out unrelated results. The search was restricted to English-language articles to maintain relevance and accessibility.

Titles and abstracts of the retrieved articles were manually screened to identify studies aligned with the research objectives. Full texts of the selected articles were then reviewed in detail to extract key information. To further enhance the search, reference lists of relevant articles were manually examined, and citation tracking was employed to identify additional studies citing pivotal articles.

This rigorous search strategy aimed to capture a complete body of literature on the topic, providing a thorough understanding of the role of antioxidants and dietary supplementation in the management of AMD.

## 3. Antioxidants in Age-Related Macular Degeneration

### 3.1. Overview of Age-Related Macular Degeneration (AMD)

#### 3.1.1. Epidemiology and Global Impact

AMD is the leading cause of irreversible blindness among the elderly in the Western world and the third leading cause worldwide [4].

AMD was estimated to affect 5.4% of the 33.6 million blind adults globally in 2020, ranking fourth among causes of blindness in individuals over 50 years, following cataracts, glaucoma, and uncorrected refractive errors [5]. Its prevalence is estimated at 8.7% in individuals aged 45–85 years, with higher rates seen in women (65% vs. 35% in men) and White populations [6].

The condition is projected to affect 288 million people by 2040 [5], with legal blindness rates in industrialized nations attributed to AMD at approximately 50% [7]. The risk of drusen, the hallmark of AMD, increases progressively with age, particularly after 60 years. The disease’s economic burden exceeds USD 340 billion, with most patients remaining ineligible for clinical treatment [8].

#### 3.1.2. Clinical Classification: Dry (Non-Exudative) and Wet (Exudative) AMD

AMD is characterized by the accumulation of extracellular deposits called drusen [9]. These deposits, appearing as white or yellow dots, form between the RPE and Bruch’s membrane due to impaired cellular debris clearance and inadequate nutrition [10].

AMD is typically classified into two forms: dry (atrophic or non-exudative) and wet (exudative or neovascular). Dry AMD is the most common form, accounting for 85–90% of cases [1]. It is characterized by the gradual degeneration of the photoreceptors, RPE cells, and choriocapillaris in the macula, [11] leading to progressive vision loss. Dry AMD progresses through three stages. Early AMD involves small (<63 µm) or a few medium-sized (<125 µm) drusen [12]. Intermediate AMD features more extensive drusen, including at least one large deposit (>125 µm), and/or pigment abnormalities. More recently, reticular pseudodrusen have been introduced as third macula risk feature of the disease, [13] associated with increased risk of progression, and incorporated in a new severity scale proposed by the AREDS and AREDS2 Research Groups [14]. Advanced dry AMD, also known as geographic atrophy (GA), is marked by progressive atrophy of the RPE and photoreceptors, often with increased visualization of the underlying choroidal vessels, causing slow but significant central vision loss [2].

Wet AMD, though less common (15% of cases), is responsible for the majority of severe vision loss associated with AMD [1]. It is characterized by macular neovascularization (MNV) development, where abnormal blood vessels penetrate Bruch’s membrane, thus further damaging the RPE and photoreceptors. These vessels are in fact prone to exudation, causing subretinal and/or intraretinal fluid, hemorrhages, and eventually macular scarring–all features variably contributing to the rapid and severe vision loss. A hallmark symptom of wet AMD is distorted vision, or metamorphopsias, where straight lines appear curved, a symptom that is historically tested by checking the Amsler’s grid. An interplay between these two forms of advanced AMD is also being constantly investigated with wet AMD sometimes arising in the setting of previous GA or, vice versa, being complicated by incident macular atrophy [15].

### 3.2. Role of Oxidative Stress in AMD Pathogenesis

#### 3.2.1. Oxidative Stress and Its Contribution to Retinal Damage

Oxidative stress, a concept introduced by Helmut Sies, refers to an imbalance between pro-oxidant and antioxidant systems in favor of the former, ultimately leading to cellular damage [16,17]. Reactive oxygen species (ROS), including superoxide anion (O_2_•^−^) and hydrogen peroxide (H_2_O_2_), are normal byproducts of mitochondrial metabolism but become harmful when produced in excess [18]. This imbalance is one of the main contributors to the pathophysiology of AMD.

The retinal oxygen-rich environment and constant exposure to light make it highly susceptible to oxidative stress [19]. In AMD, oxidative stress is well known to be closely linked to the degeneration of RPE cells. These cells are responsible for phagocytosing the photoreceptor outer segments (POS), a process that generates ROS [20]. If ROS levels exceed the RPE’s antioxidant capacity, they can trigger further oxidative damage, impairing cellular structures and promoting RPE cell death.

Additionally, ROS-induced damage disrupts vascular signaling pathways, contributing to vascular dysfunction, a hallmark of many retinal diseases [21].

#### 3.2.2. Accumulation of Reactive Oxygen Species (ROS) and Retinal Aging

With age, the retina experiences an accumulation of oxidative stress markers, including lipofuscin (a photoinducible ROS generator), 8-oxoguanine (oxidative DNA damage product), mtDNA damage, and lipid peroxidation products like 4-Hydroxynonenal (4-HNE) and Malondialdehyde (MDA) [22]. These changes are especially pronounced in the RPE, composed of postmitotic cells incapable of regeneration [23]. Over time, accumulated damage reduces RPE cell density, leaving remaining cells to cope with higher ROS levels, further amplifying oxidative stress and promoting retinal pathology [24,25].

The “free radical hypothesis”, proposed by Harman in 1956, [26] suggests that endogenous free radicals cause cumulative macromolecular damage, leading to aging and reduced physiological function [27]. In the retina, aging increases ROS production due to electron leakage from the mitochondrial electron transport chain, combined with a decline in antioxidant defenses. This imbalance exacerbates structural damage in the retina, particularly in the posterior pole, where lipid peroxidation becomes more prevalent [16,17].

This hypothesis emphasizes that narrowing the gap between ROS production and antioxidant defenses could reduce structural damage, slow aging-related changes, and preserve retinal health [28].

### 3.3. Pathophysiology of Oxidative Damage in AMD

#### 3.3.1. Retinal Anatomy and Susceptibility

##### High Metabolic Activity and Oxygen Consumption of the Retina

The retina is one of the most oxygen-demanding tissues in the human body, reflecting its high metabolic activity [29]. The outer retina primarily relies on oxygen supplied by the choroidal circulation, while the inner retina mostly depends on the retinal vasculature [30]. Notably, the most central part of the macula–the foveola (350–500 μm diameter)–lacks retinal vessels and relies solely on the choriocapillaris for oxygen and nutrients, exposing the RPE to elevated oxygen levels that promote the production of ROS like O_2_•^−^, •OH, and H_2_O_2_ [31].

**Figure 1:**
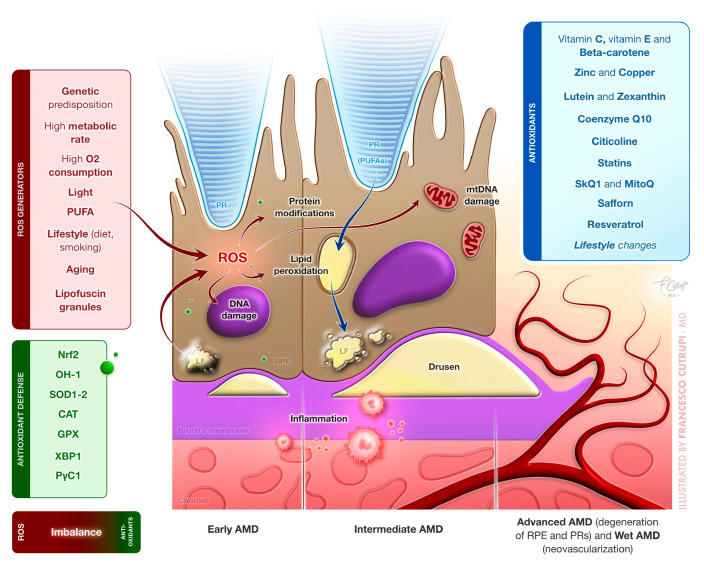
Representative image of oxidative stress mechanisms leading to development of age-related macular degeneration (AMD) and antioxidants molecules potentially contributing to its prevention or slowing its progression.

Photoreceptors, with their exceptionally high metabolic activity, require significant amounts of oxygen and nutrients delivered via blood vessels. This intense oxygen consumption makes the oxygen supply to the retina among the highest in the body, contributing to its unique vulnerability to oxidative stress (Figure 1) [32].

##### Photoreceptor-RPE Complex as a Target of Oxidative Damage

Photoreceptors are uniquely susceptible to oxidative stress due to their constant exposure to light and oxygen [33]. High-energy light, concentrated in the macula, further intensifies oxidative damage. POS, rich in polyunsaturated fatty acids (PUFAs) like Docosahexaenoic Acid(DHA) are highly prone to lipid peroxidation, producing toxic byproducts such as malondialdehyde (MDA) and 4-hydroxy-2-nonenal (4-HNE) [34,35]. These reactive aldehydes can damage cellular proteins, DNA, and membranes, leading to inflammation and cell dysfunction.

Each night, approximately 10% of POS are shed, and the RPE phagocytoses and degrades their debris. This process is critical for photoreceptor renewal and function, but oxidative damage to the RPE impairs this maintenance, often leading to secondary photoreceptor death [36].

In AMD, oxidative stress is indeed a key driver of RPE degeneration and inflammation. Dysregulation of the complement pathway and the accumulation of oxidative byproducts, such as carboxyethylpyrrole (CEP) formed from DHA, contribute to drusen formation, geographic atrophy, and vision loss [37].

### 3.4. Molecular Mechanisms of Oxidative Stress

#### 3.4.1. Lipofuscin Accumulation

Lipofuscin, a pigment complex composed of lipids, proteins, and derivatives like A2E, accumulates progressively in dysfunctional RPE cells. This accumulation is a key feature of aging and various retinal diseases including AMD [38].

The photoreactivity of lipofuscin is primarily responsible for generating ROS such as singlet oxygen, superoxide anion, and hydrogen peroxide (H_2_O_2_) under aerobic conditions. These ROS contribute to lipid peroxidation, damage to cellular components, and the inactivation of antioxidant enzymes like superoxide dismutase (SOD) and catalase, leading to RPE dysfunction [39,40].

#### 3.4.2. Drusen Formation and the Role of Oxidative Stress in Their Development

Drusen, extracellular deposits located between the RPE basal lamina and the Bruch’s membrane inner collagenous layer, are believed to form when RPE cells fail to effectively store shed POS or degrade cellular debris [41]. Oxidative stress plays a key role in this process, contributing to RPE degradation and drusen accumulation [42].

Proteomic analyses conducted on drusen have identified numerous proteins linked to AMD, including βB1-crystallin, Clusterin (APOJ), Complement Component 9 (CC9), αB-crystallin, TIMP3, Vitronectin, and Annexin 2. Notably, drusen from AMD patients also contain proteins associated with immune activation, such as β-amyloid, C-reactive protein (CRP), and the membrane attack complex (MAC), highlighting their role in triggering inflammation [42,43].

Drusen negatively impact retinal health in two ways: they stimulate chronic inflammation and impair the exchange of oxygen and nutrients between Bruch’s membrane and RPE cells [44,45,46].

#### 3.4.3. P2X7 Receptor and Oxidative Stress

The P2X7 receptor plays a crucial role in the pathology of age-related macular degeneration (AMD), particularly through its involvement in oxidative stress, inflammation, and cell death. Amyloid β, a primary component of drusen, induces oxidative stress and apoptosis, [47,48] with its toxicity linked to P2X7 activation in AMD models [49]. Evidence suggests a pivotal role of the P2X7 receptor-pannexin-1 complex in mediating oxysterol toxicity in retinal cells [50]. Activation of the P2X7 receptor leads to the formation of large, non-selective membrane pores, triggering inflammasome activation, oxidative stress, and apoptosis [51]. Additionally, increased surface expression of the P2X7 receptor stimulates IL-1β maturation and secretion, with studies demonstrating that inhibition of P2X7 or IL-1β significantly reduces photoreceptor apoptosis and inflammation in AMD-related models [52].

Furthermore, recent advancements in elucidating the structure of the P2X7 receptor’s intracellular domain offer opportunities for developing novel P2X7 antagonists. Blocking the P2X7 receptor could inhibit inflammatory pathways, regulate growth factor expression, and modulate proteins involved in cell-cell communication, presenting a promising therapeutic avenue for AMD management [53].

#### 3.4.4. Mitochondrial Dysfunction in Retinal Pigment Epithelium (RPE)

Mitochondria are a primary source of ROS and are particularly vulnerable to oxidative stress [54]. ROS can damage mitochondrial DNA (mtDNA), leading to alterations of essential proteins for mitochondrial respiration [55]. Unlike nuclear DNA, mtDNA is more susceptible to oxidative damage due to its proximity to ROS production sites, lack of protective histones, absence of introns, and less efficient repair mechanisms. This heightened vulnerability leads to the rapid accumulation of mutations in mtDNA, impairing the respiratory chain and further exacerbating ROS production [56,57].

Mitochondrial dysfunction in RPE cells is associated with decreased ATP production, reduced mitochondrial membrane potential, altered calcium dynamics, and increased oxidative stress. For example, a deficiency in manganese superoxide dismutase (MnSOD), a key mitochondrial antioxidant enzyme, has been shown to amplify mitochondrial oxidative stress. This triggers superoxide anion accumulation, apoptotic cell death, RPE degeneration, Bruch’s membrane thickening, and disorganization of photoreceptor outer and inner segments. These mitochondrial impairments are critical contributors to retinal degeneration and diseases like AMD [58].

### 3.5. Genetic and Environmental Risk Factors

#### 3.5.1. Variants in the Complement Factor H (CFH) Gene and Oxidative Stress

The complement system is an integral part of the immune response, involving over 40 proteins and receptors across three pathways: classical, lectin, and alternative. Factor H (CFH) is the primary negative regulator of the alternative pathway, protecting host tissues from complement-mediated damage [59]. CFH is a 155 kDa protein with 20 short consensus repeats (SCRs), abundant in human serum [60].

The CFH gene, located on chromosome 1q31, has been strongly linked to AMD. A key finding from a 2005 genome-wide association study (GWAS) identified an intronic variant in CFH, specifically the Y402H polymorphism, [61] where tyrosine (Y) is replaced by histidine (H) at position 402. This polymorphism lies in a region that binds to heparin and C-reactive protein (CRP), with elevated CRP levels associated with AMD. Studies estimate that this variant accounts for up to 53% of the population’s risk for late AMD [62]. Interestingly, a recent study by Schwartz et al. identified a suggestive –albeit not significant– link between CFH gene and the quantitative RPD load [63].

CFH linkage disequilibrium gives rise to several haplotypes, with the most common haplotype (H1) strongly associated with AMD risk (odds ratio: 2.46) [64]. A rare but highly penetrant variant, R1210C, was later identified in SCRs 19–20, another CFH region implicated in advanced AMD and extensive drusen accumulation [65].

In AMD, CFH risk variants contribute to excessive complement activation and deposition in the choroidal capillaries and vessels. This leads to plasma protein leakage into Bruch’s membrane, exacerbating oxidative stress and inflammation, both central to AMD pathogenesis [61].

#### 3.5.2. Risk Factors

Aging is the strongest risk factor for AMD, with additional modifiable contributors including smoking, diet, high body mass index, serum cholesterol, and sunlight exposure [66,67].

Smoking induces ROS formation through nicotine, cadmium, and hydroquinone, causing oxidative damage to the retina [68]. Cadmium accumulates in the RPE and choroid, while nicotine promotes nitric oxide production and angiogenesis [69,70]. Smoking damages mitochondria, increases lipid peroxidation, and induces RPE cell death. Studies, including the Blue Mountains Eye Study and AREDS cohort, link smoking to GA development, with cessation reducing AMD risk [71].Blue light exposure amplifies oxidative stress by damaging photoreceptor segments and RPE cells, inducing apoptosis and DNA damage through photooxidation of lipofuscin and reactive photoproducts like A2E [72,73]. The enzyme heme oxygenase-1 (HO-1), linked to oxidative stress, is upregulated in light-damaged retinas [74].

### 3.6. Antioxidants in AMD

The progressive understanding of the role of oxidative stress in macular degeneration, combined with the heightened vulnerability of this tissue to such damage, has prompted the suggestion of various antioxidants to help mitigate the progression of AMD:

#### 3.6.1. Antioxidants of Interest

##### Vitamin C, Vitamin E and Beta-Carotene

A powerful antioxidant, Vitamin C protects critical biomolecules—including proteins, lipids, carbohydrates, and nucleic acids—from damage caused by oxidants generated during metabolism and exposure to environmental toxins such as cigarette smoke [75]. It regulates gene expression, prevents oxidative damage to DNA and proteins, and acts as a cofactor for enzymes like dopamine β-monooxygenase and prolyl 4-hydroxylase [76]. Its strong reducing properties, derived from the lactone ring’s hydroxyl groups, enable it to neutralize free radicals, protecting cellular components from oxidation [77].

Vitamin E: All forms of Vitamin E function as potent antioxidants by neutralizing lipid peroxyl radicals through hydrogen donation. Natural forms, such as γ-tocopherol (γT), uniquely trap reactive nitrogen species, which are elevated during inflammation [78,79]. Vitamin E also inhibits the generation of inflammatory mediators like prostaglandins and leukotrienes, providing broad cellular protection [80].

Beta-carotene, a precursor to retinol (Vitamin A), is a potent antioxidant known for its ability to mitigate free radical damage by neutralizing reactive oxygen species (ROS). This capability arises from its unique molecular structure, characterized by conjugated double bonds, [81] which play a critical role in energy transfer reactions and the quenching of singlet oxygen [82]. Beta-carotene has been found to efficiently reduce harmful radicals, such as trichloromethylperoxyl radicals, and inhibit the oxidation of model compounds like tetralin and methyllinoleate [83]. Widely found in fruits and vegetables, beta-carotene also exhibits potential antineoplastic and chemopreventive effects [84].

##### Zinc and Copper

Zinc (Zn) exhibits significant antioxidant properties, largely independent of its role in zinc metalloenzyme activity. It reduces oxidative stress by inducing metallothionein synthesis and stabilizing sulfhydryl groups. Chronic zinc supplementation has been shown to enhance metallothionein levels across various organs, which may act as ultimate antioxidants [85,86]. Zinc also protects δ-aminolevulinate dehydratase from oxidative inactivation by preventing thiol oxidation and disulfide bond formation [87]. Furthermore, it mitigates site-specific oxidative injuries and reduces hydroxyl radical (·OH) formation by antagonizing redox-active transition metals. Additionally, zinc increases CuZn superoxide dismutase (CuZnSOD) activity, further enhancing its antioxidant capabilities [88].

Copper (Cu) serves as a critical cofactor in CuZnSOD, playing a pivotal role in oxidative stress regulation [89]. Copper supplementation has demonstrated antioxidant effects, such as protecting red blood cells (RBCs) from oxidative damage [90,91]. However, an imbalance in copper and zinc levels can heighten susceptibility to oxidative damage, underscoring the importance of their equilibrium in maintaining redox stability [90,91].

##### Lutein and Zeaxanthin

Lutein and zeaxanthin, xanthophyll carotenoids concentrated in the macula, provide critical protection for photoreceptors through their dual roles as light filters and antioxidants [92]. These macular pigments absorb up to 90% of harmful blue light, reducing photochemical damage, and quench singlet oxygen, preventing light-induced lipid peroxidation in retinal cells [93]. Beyond their retinal effects, lutein supplementation has been found to lower key factor D secretion in the alternative complement activation pathway, helping to reduce inflammation and support AMD prevention [94].

##### Coenzyme Q10

Coenzyme Q10 (CoQ10) plays a significant role in protecting the retina from oxidative stress by inhibiting ROS production and preventing neuroretinal cell damage. It is an essential cofactor of the electron transport chain and helps maintain mitochondrial membrane potential [95]. Studies have demonstrated its effectiveness in improving visual function in patients with early AMD. CoQ10 enhances retinal cell viability, reduces apoptosis caused by UV and γ-radiation, and protects retinal layers from UV-induced apoptosis, even when administered as eye drops [96]. A combination of CoQ10 with acetyl-L-carnitine (ALC), PUFAs, and vitamin E has shown sustained improvements in retinal function over 24 months [97]. Another study by Dongwook Lee and colleagues demonstrated how, in mice models, CoQ10 ameliorated glutamate toxicity and decreased oxidative stress-mediated retinal ganglion cell degeneration in the retina [98]. Additionally, idebenone, a synthetic analog of CoQ10, has demonstrated cytoprotective and antiapoptotic effects in RPE cells by reducing ROS, stabilizing the BAX/Bcl-2 ratio, and mitigating oxidative stress-induced apoptosis [99].

##### Citicoline

Citicoline, a mononucleotide comprising ribose, cytosine, pyrophosphate, and choline, has shown potential antioxidant and cytoprotective effects [100]. It plays a role in glutathione synthesis, which reduces lipid peroxidation in the central nervous system [101]. Sonali Nashine and colleagues demonstrated that Citicoline treatment downregulated pro-apoptotic genes such as BAX, Caspase-3, and Caspase-9 in AMD RPE cybrid cells, significantly reducing their expression levels by 28.6%, 77.2%, and 37.2%, respectively [102]. It also decreased reactive oxygen species (ROS) levels by 22.8% while upregulating antioxidant genes HMOX1 and HMOX2 [102]. Additionally, Citicoline reduced the expression of HIF-1α (a hypoxia marker) by 34% and VEGF (an angiogenesis marker), contributing to its cytoprotective effects in AMD-related oxidative stress [102].

##### Vitamin D3

Several studies have explored the potential role of vitamin D3 in age-related macular degeneration (AMD), focusing on its serum levels and its anti-oxidative and anti-angiogenic properties. Research indicates that higher serum vitamin D3 levels are inversely associated with early AMD, suggesting that vitamin D3 may help in the management or progression reduction of the disease. For example, one study found that individuals in the highest quintile of serum vitamin D had significantly lower odds of early AMD (OR = 0.64) [103]. A meta-analysis by Cedric Annweiler and colleagues further supported this, showing that lower circulating 25-hydroxyvitamin D (25OHD) levels were associated with an increased risk of late-stage AMD, with concentrations under 50 nmol/L being particularly linked to late AMD (OR = 2.18) [104]. 

Additionally, animal models and cell studies have shown that vitamin D3 can reduce retinal neovascularization and oxidative stress [105]. In a mouse model of ischemic retinopathy, calcitriol, an active form of vitamin D3, significantly inhibited retinal neovascularization [105]. In another study based on a rat model, vitamin D3 (VD3) reduced inflammation by lowering myeloperoxidase (MPO) activity, ROS production, and inflammatory markers like TNF-alpha, iNOS, and COX-2. VD3 also decreased edema, neutrophil activity, and pain, highlighting its anti-inflammatory and antioxidative potential [106].

Lazzara F et al. in their study involving human retinal pigmented epithelial (RPE) cells, researchers pretreated the cells with 1,25(OH)_2_D_3_ (50 nM) for 24 h before exposing them to hydrogen peroxide (H_2_O_2_) to induce oxidative stress, mimicking the conditions seen in age-related macular degeneration (AMD). When the cells were allowed to recover for an additional 24 h, the treatment with 1,25(OH)_2_D_3_ significantly restored cell viability. Moreover, vitamin D3 treatment notably reduced the expression of key inflammatory markers such as MMP-9, IL-1β, and TNF-α, suggesting that vitamin D3 effectively modulates the inflammatory response and oxidative stress [107].

##### Curcumin

Curcumin, a major bioactive component of turmeric (Curcuma longa), has been studied as a potential adjuvant in the management of age-related macular degeneration (AMD) due to its multifaceted biological properties. It modulates various cell signaling pathways and interacts with molecular targets involved in cell cycle regulation, apoptosis, proliferation, angiogenesis, and inflammation. Curcumin strongly upregulates heme oxygenase 1 (HO-1) activity, a protective enzyme with anti-inflammatory, antioxidant, anti-apoptotic, and anti-proliferative effects [108]. Through activation of the Nrf2/HO-1 signaling pathway, which involves the ERK pathway, curcumin provides significant protection against damage to retinal pigment epithelial (RPE) cells induced by oxidative stress [109]. It reduces the expression of oxidative stress biomarkers, including superoxide dismutase (SOD) and glutathione, and mitigates apoptosis in aged RPE cells, thereby improving cell viability and reducing oxidative damage [110]. Additionally, curcumin has been shown to significantly lower protease-mediated retinal ganglion cell (RGC) and amacrine cell death at a dosage of 10 μM in vivo [110]. Furthermore, curcumin’s role in reducing AMD progression has been supported by studies indicating a reduced risk of nonexudative and advanced AMD with its use [111].

##### Statins

Studies suggest that statins may offer protective benefits in AMD. High-dose atorvastatin has been shown to promote the regression of drusen deposits and improve visual acuity (VA) by +3.3 letters in patients with intermediate AMD, with no progression to advanced neovascular AMD [112]. Furthermore, statin use in early AMD reduces risk by approximately 17% (RR, 0.83; 95% CI, 0.66–0.99), and it offers protective effects against late-stage exudative AMD (RR, 0.90; 95% CI, 0.80–0.99) but not geographic atrophy [113].

The protective effects of statins extend beyond cholesterol reduction. They modulate oxidative stress by stimulating the Nrf2(nuclear factor erythroid 2-related factor 2)/HO-1 signaling pathway, enhancing cellular defense mechanisms [114]. Statins increase Nrf2 DNA-binding activity and upregulate antioxidant enzymes like HO-1 and GPX while suppressing oxidant enzymes such as NAD(P)H oxidase and myeloperoxidase [114]. Additionally, they improve endothelial function by enhancing nitric oxide bioavailability [115].

The Antioxidants, along with their proposed mechanism of action and effect in the setting of AMD have been summarized in Table 1.

### 3.7. Landmark Trials: The AREDS Studies

#### 3.7.1. AREDS 1: Study Design, Results, and Impact on AMD Management

The Age-Related Eye Disease Study (AREDS) was a long-term, multicenter, prospective trial involving 4757 participants aged 55–80 years [116]. Funded by the National Eye Institute, AREDS aimed to study the progression and risk factors of AMD and cataracts, while testing the efficacy of high-dose vitamin and mineral supplements in AMD management [117]. The study developed a grading system for AMD lesions, focusing on advanced AMD outcomes such as neovascular AMD and central geographic atrophy.

The original AREDS formula included antioxidants (vitamins C and E, beta-carotene) and zinc, chosen for their systemic benefits and potential to slow AMD progression [116]. Results showed a 25% reduction in advanced AMD risk in patients with intermediate or advanced disease, highlighting its moderate but significant public health impact [84]. The formulation has also shown to slow Geographic Atrophy progression toward the central macula. The proposed mechanism is by augmenting the natural phenomenon of foveal sparing [118].

Further analysis revealed that higher dietary intake of lutein, zeaxanthin, and omega-3 fatty acids also reduced AMD risk. These findings led to AREDS-2, which explored modifying the original formula by adding lutein, zeaxanthin, and omega-3s. Notably, omega-3 consumption (e.g., from fish) was linked to a 25% reduced risk of AMD progression, reinforcing the role of dietary factors in AMD management [119].

#### 3.7.2. AREDS 2: Modified Formulation and Updated Findings

The AREDS2 study, launched by the National Eye Institute in 2006, aimed to enhance the original AREDS formula by evaluating the addition of omega-3 fatty acids (DHA and EPA) and carotenoids lutein and zeaxanthin, while removing beta-carotene and reducing zinc levels [120]. The removal of beta-carotene was due to growing concerns regarding the carotenoid increasing the risk of lung cancer in smokers [121]. This five-year trial involved over 4000 participants with intermediate AMD and sought to assess whether these changes could further reduce the progression to advanced AMD [120].

The primary analysis found no overall additional reduction in advanced AMD risk with the inclusion of omega-3s or lutein and zeaxanthin [120]. However, a secondary analysis revealed that replacing beta-carotene with lutein and zeaxanthin led to significant benefits, including an 18% reduction in the risk of advanced AMD progression and a 22% decrease in neovascular AMD risk [122].

Additionally, reducing zinc from 80 mg to 25 mg showed no significant loss of efficacy, and the removal of beta-carotene improved lutein and zeaxanthin absorption. Omega-3 fatty acids, however, did not show benefits for patients with advanced AMD. These findings support the updated AREDS2 formulation as a safer and equally effective alternative to the original [122].

#### 3.7.3. Critique of the AREDS Formulations (Benefits, Limitations and Controversies)

AREDS 1: While it demonstrated modest success in reducing AMD progression, concerns arose regarding zinc’s potential genitourinary complications and a lack of long-term safety data. Some investigators questioned the study’s funding and noted the absence of data replication. Additionally, beta-carotene in the formulation increased lung cancer risk for current or former smokers, prompting interest in substituting it with lutein and zeaxanthin [123].

AREDS 2: This study sought to address these concerns but faced its own challenges. Its complex design, involving secondary randomization, may have affected the evaluation of lutein/zeaxanthin and omega-3 fatty acids (DHA and EPA) [122]. The primary analysis did not conclusively show benefits or harms of these additions, but secondary analyses suggested lutein and zeaxanthin reduced the risk of advanced AMD. However, omega-3 supplementation showed no effect, possibly due to the less bioavailable ethyl ester form used [124].

AREDS 2 lacked a true control group, as most participants continued the original AREDS formula, and dietary habits varied widely. Additionally, the study population—primarily well-nourished white females—was not representative of broader demographic diversity, limiting generalizability [123]. Many participants already consumed nutrient-rich diets or supplements, further complicating findings. Despite these challenges, AREDS 2 provided insights into refining AMD management strategies [125].

### 3.8. Mechanisms of Action of Antioxidants in AMD

#### 3.8.1. Direct Scavenging of Free Radicals

Antioxidants neutralize reactive oxygen species (ROS) in retinal tissues through non-enzymatic reactions, where small antioxidant molecules either donate or accept electrons to stabilize free radicals. Ascorbate (Vitamin C) is a key antioxidant, and its oxidized form, dehydroascorbic acid (DHA), can be quickly reduced back to ascorbate within cells by systems like glutathione (GSH) [126]. GSH not only directly interacts with ROS and electrophiles but also donates electrons to GSH-dependent enzymes, such as glutathione peroxidases (GPXs) and glutathione transferases (GSTs) [127]. Additionally, Vitamin E quenches ROS by transferring a phenolic hydrogen atom to the oxidant, and the resulting tocopherol radical can be regenerated by ascorbate [128].

#### 3.8.2. Modulation of Inflammatory Pathways

Antioxidants play a crucial role in reducing inflammation and complement activation by modulating key signaling pathways. Melatonin, for example, can enhance the expression of antioxidant enzymes by activating the Nuclear factor erythroid-derived 2-like 2 (NRF2) transcriptional pathway while simultaneously downregulating prooxidant enzymes such as lipoxygenases and nitric oxide synthases (NOS), which contribute to ROS production [129]. The KEAP1-NRF2 pathway is a master regulator of the antioxidant response, and its activation boosts cellular antioxidant capacity, making it significant in various ocular diseases [130]. Lutein treatment, for instance, has been shown to upregulate mRNA levels of superoxide dismutase (SOD) enzymes in the photo-stressed RPE choroid, enhancing SOD activity in treated mice. Additionally, overexpressing NRF2 in the Pde6b mouse model improved RPE function and alleviated cone photoreceptor damage by activating oxidative defense pathways, including glutathione synthesis [131]. Lutein treatment also suppressed macrophage recruitment, a key factor in AMD pathogenesis, by reducing mRNA levels of MCP-1, a macrophage-recruiting factor [132]. Furthermore, sulforaphane, an isothiocyanate from cruciferous vegetables, has demonstrated therapeutic effects in retinal degeneration models by activating NRF2, which upregulates antioxidant proteins like thioredoxin (TXN), thioredoxin reductase (TXNRD), and heme oxygenase-1 (HO-1) [133].

#### 3.8.3. Increasing Macular Pigment Optical Density

Antioxidants, particularly lutein and zeaxanthin, play have been studied for their role in increasing macular pigment optical density (MPOD), which is vital in protecting the macula from photooxidative damage. These carotenoids, the primary components of macular pigment, exert protective effects through their antioxidant and light-screening properties, reducing the risk of age-related macular degeneration (AMD) [134]. Studies have shown a significant inverse relationship between central MPOD levels and AMD risk factors such as age, tobacco use, and family history, suggesting that a deficiency in macular pigment may contribute to an increased risk of AMD [135]. Supplementation with lutein and/or zeaxanthin, ranging from 2–30 mg per day or through a high carotenoid diet, has consistently demonstrated a rise in macular carotenoids and MPOD [136,137,138,139,140,141,142].

For instance, Richard A. Bone and colleagues reported a significant increase in MPOD in subjects supplemented with 20 mg/day of predominantly meso-zeaxanthin, compared to a decrease in the placebo group [143]. However, the bioavailability of lutein may be reduced by polyunsaturated fatty acids (PUFA), potentially diminishing its benefits on MPOD and contrast sensitivity (CS). Notably, lutein-only supplementation has shown significant improvements in MPOD and CS, whereas combined lutein and PUFA supplementation showed less pronounced effects [144].

A network meta-analysis of randomized controlled trials by Welli Hu and colleagues highlighted that various antioxidant combinations significantly improved MPOD and visual outcomes. Specifically, the combination of lutein, zeaxanthin, and fatty acids was most effective for enhancing MPOD and ranked highly for improving contrast sensitivity and photostress recovery time, underscoring the potential of tailored antioxidant regimens in mitigating AMD progression [145].

### 3.9. Novel Antioxidants and Emerging Therapies

#### 3.9.1. Mitochondrial-Targeted Antioxidants

Mitochondrial-targeted antioxidants, such as SkQ1 and MitoQ, are emerging strategies aimed at protecting mitochondrial function. SkQ1 is a conjugate of plastoquinone with a lipophilic decyltriphenylphosphonium cation, designed to target mitochondria and mitigate oxidative damage. This compound has shown promise in protecting against diseases associated with mitochondrial dysfunction [146,147]. The NLRP3 inflammasome, linked to various diseases, plays a role in the therapeutic action of these antioxidants by modulating inflammatory responses [148]. MitoQ, specifically designed to reduce mitochondrial ROS (mtROS), has been studied in clinical trials for its potential to protect against oxidative damage, though it has not yet been FDA-approved [149].

In animal studies, SkQ1 has shown significant protective effects in retinopathy models. When introduced to OXYS rats at 1.5 months of age, SkQ1 completely prevented early signs of retinopathy, improving retinal pigment epithelium (RPE) function and reducing lipofuscin accumulation [147,150]. These findings suggest that early retinopathy, such as in AMD, is linked to decreased VEGF expression in the retina, and SkQ1 may help restore this mechanism by enhancing RPE function. Additionally, SkQ1’s therapeutic action includes the restoration of mitochondrial structure and function, leading to the reversal of retinal pigment epithelium insufficiency [146,151]. Importantly, SkQ1 does not show negative effects in control rats, indicating its safety as a treatment option [147,152].

#### 3.9.2. Nanoceria Particles

Cerium oxide nanoparticles (nanoceria) have emerged as promising agents in the treatment of age-related macular degeneration (AMD) due to their catalytic ability to scavenge reactive oxygen species (ROS). By mimicking the enzymatic activities of superoxide dismutase and catalase, nanoceria effectively reduce oxidative stress—a key factor in AMD pathogenesis [153]. Studies in animal models have demonstrated that nanoceria exhibit long-lasting efficacy without causing collateral damage to retinal structures [154].

Fluorescein-isothiocyanate-labeled nanoceria (FITC-CeO2) have been observed accumulating in the outer retina, specifically within the photoreceptor outer segments and retinal pigment epithelium (RPE) cells. This localization indicates that nanoceria may target the outer retina by enhancing RPE protection, which is hypothesized to be a primary mechanism underlying their therapeutic effects [155,156].

In an in vivo study by Xiaohong Zhou et. al, a single intravitreal injection of nanoceria into a mouse model significantly inhibited ROS elevation in the retina. This treatment reduced vascular endothelial growth factor (VEGF) expression in the photoreceptor layer and mitigated both intraretinal and subretinal neovascular lesions [153]. Another investigation revealed that administering nanoceria at postnatal day 28 in Vldlr null mice led to the suppression of various pro-inflammatory cytokines and pro-angiogenic factors, such as Vegfa, Fgf1, and Fgf2, while upregulating anti-angiogenic genes within one week [157].

Annamaria Tisi and her team studied a rat model of acute light damage (LD), which shares many features with AMD. Intravitreal administration of CeO2 nanoparticles three days before light exposure effectively prevented RPE cell death and degeneration. Fluorescently labeled nanoceria localized within RPE cell cytoplasm, where they inhibited epithelial-mesenchymal transition and modulated autophagy by downregulating LC3B-II and p62. Additionally, CeO2 nanoparticles prevented nuclear localization of LC3B, demonstrating their potential as a therapeutic strategy to counteract RPE degeneration [158].

Recent advancements in nanoceria delivery systems further enhance their therapeutic promise. Alginate-gelatin hydrogels loaded with oligochitosan-coated cerium oxide nanoparticles (OCCNPs) have shown biocompatibility and efficacy in preventing apoptosis, angiogenesis, and inflammation in AMD cellular models [159]. Moreover, glycol chitosan-coated cerium oxide nanoparticles (GCCNPs) delivered into nuclear factor erythroid 2-related factor (Nrf2) knockout mice, exposed to mild white light, protected against progressive retinal oxidative damage. The combination of GCCNPs with an alginate-gelatin hydrogel produced synergistic antioxidant effects, accelerating recovery of RPE and photoreceptor cells [160].

#### 3.9.3. Gene Therapy

Gene therapy has gained significant attention as a potential treatment for age-related macular degeneration (AMD), particularly in its wet form [161]. The goal of gene therapy in AMD is to provide a long-lasting solution by enabling the eye to produce its own therapeutic proteins. Several gene therapy products have been developed for this purpose, including AVA-101, which uses an AAV2 vector to deliver a soluble human VEGF receptor 1, and 119ADVM-022, which contains an AAV.7m8 capsid with an aflibercept expression cassette. RGX-314, another promising candidate, uses an AAV8-based vector to express a monoclonal antibody fragment that binds to VEGF-A, thus suppressing neovascularization [161]. These therapies aim to reduce the need for frequent anti-VEGF injections and provide long-term relief for wet AMD [161].

An emerging area of gene therapy research is its potential as an antioxidant strategy for treating dry AMD, a chronic condition often driven by oxidative stress and retinal cell damage. The ability of gene therapy to target the retina and retinal pigment epithelium (RPE) offers the possibility of long-term protection against oxidative damage, which could be crucial for managing advanced dry AMD [162].

One notable study by Biswal et al. investigated the potential of antioxidant gene therapy in a murine model of RPE atrophy. By inducing mitochondrial oxidative stress in the RPE through the conditional deletion of the Sod2 gene (which encodes manganese superoxide dismutase, MnSOD), they were able to restore Sod2 expression in the RPE using adeno-associated virus (AAV) vectors. Their results showed that the AAV-Sod2 treatment significantly reduced oxidative stress, as evidenced by a 54% reduction in nitrotyrosine levels in treated eyes compared to controls. Furthermore, electroretinography (ERG) assessments revealed significant improvements in retinal function, with treated mice showing up to 80% better a-wave responses and more than a twofold increase in c-wave amplitudes. These findings suggest that early delivery of antioxidant gene therapy, such as AAV1-Sod2, could help preserve retinal function and prevent the progression of dry AMD [162].

Another promising therapeutic approach is GT-005, developed by Gyroscope Therapeutics (a Novartis company). GT-005 uses an AAV2 capsid to deliver a gene cassette encoding complement factor I (CFI), a natural inhibitor of the complement system, which plays a key role in the progression of advanced AMD. By inducing sustained expression of CFI in target cells, GT-005 aims to downregulate the complement system and mitigate its contribution to AMD pathogenesis [163].

In addition, Janssen’s JNJ-81201887 utilizes an AAV2 vector to increase the expression of sCD59, an anti-inflammatory protein that regulates the complement pathway. In patients with neovascular AMD (nAMD) and geographic atrophy (GA), sCD59 is under-expressed, contributing to inflammation and retinal damage [164]. A clinical trial (NCT03144999) [165] demonstrated that a single intravitreal injection of JNJ-81201887 was well tolerated and led to a sustained reduction in GA lesion growth, with the high-dose cohort showing a decline in lesion progression over a 24-month period [164].

These studies highlight the growing potential of gene therapy not only as a means to address the core mechanisms of AMD, such as neovascularization and inflammation, but also as a strategy to combat oxidative stress, one of the key drivers of retinal damage in dry AMD.

#### 3.9.4. Saffron

Several clinical studies have evaluated the impact of saffron supplementation (20–50 mg daily) on age-related macular degeneration (AMD), showing improvements in vision-related parameters. Despite variations in study designs, saffron consistently enhanced visual function. The active compounds in saffron, including crocins, crocetin, picrocrocin, and safranal, exhibit strong antioxidant properties that protect biomolecules from free radical damage.

In a study by Benedetto Fasini and colleagues, 25 AMD patients were given saffron or placebo for three months, demonstrating significant improvements in retinal flicker sensitivity, suggesting a potential role for saffron in AMD treatment beyond antioxidants [166]. A follow-up study with 29 early AMD patients over 14 months showed improvements in macular function and visual acuity, with a two-line increase in Snellen visual acuity [167].

Saffron’s mechanisms include modulating gene expression, regulating calcium signaling through P2X7 receptors, and influencing the endocannabinoid system, offering neuroprotective effects in retinal cells [168]. By targeting the P2X7 receptor, saffron may regulate calcium signaling, preventing the formation of large non-selective membrane pores and thereby reducing inflammasome activation and subsequent inflammatory cascades [53]. Additionally, by influencing the endocannabinoid system, saffron may help restore cellular homeostasis and promote the survival of photoreceptors and retinal pigment epithelial cells [168]. These multifaceted mechanisms position saffron as a promising therapeutic agent for managing AMD and protecting against the degeneration of retinal structures.

#### 3.9.5. Resveratrol

Resveratrol, a natural phenolic compound found in foods like grapes, peanuts, and red wine, is known for its antioxidant, anti-inflammatory, and immunomodulatory effects. It helps improve neuronal activity by restoring endothelial function and reducing reactive oxygen species (ROS) production [169]. Resveratrol also upregulates the expression of antioxidant and anti-aging genes, while lowering ROS and inflammatory markers [170].

It reduces levels of nitric oxide (NO), inhibits lipid peroxidation, and increases the ratio of reduced to oxidized glutathione (GSH). Furthermore, it enhances the activities of antioxidants like catalase (CAT) and superoxide dismutase (SOD) [171,172]. Resveratrol also decreases pro-inflammatory cytokines such as IL-1β, IL-8, TNF-α, and MCP-1, and it promotes the activation of Nrf2, a key regulator of antioxidant responses [173]. These combined actions make resveratrol a promising compound for protecting retinal cells from oxidative stress and inflammation.

### 3.10. Challenges with the Use of Antioxidants in AMD Management

#### 3.10.1. Variability in Patient Responses (Genetic Polymorphism Affecting Treatment Response)

Genetic factors, particularly polymorphisms in the CFH and ARMS2 genes, can influence how patients with AMD respond to antioxidant and zinc treatments. Studies by Awh and colleagues suggest that individuals without CFH risk alleles and with ARMS2 risk alleles benefit most from zinc-only supplementation. Conversely, patients with CFH risk alleles but no ARMS2 risk alleles respond better to antioxidant-only therapy, while zinc supplementation may accelerate AMD progression in this group [174].

Supporting these findings, another study showed that patients with two CFH risk alleles experienced more progression with zinc treatment than with placebo. However, individuals with fewer CFH risk alleles and one or two ARMS2 risk alleles benefited from zinc and antioxidant treatments [175].

While Awh et al. highlight a potential pharmacogenomic approach to supplementation, Assel and colleagues found no strong evidence for genotype-based treatment strategies. Assel et al. re-evaluated the data, identifying errors and adjusting for multiple testing, which led to the conclusion that genotype-treatment interactions were not statistically significant [176]. Their independent analysis on a separate dataset yielded negative results, challenging the claim that genetic variations significantly alter treatment efficacy. This discrepancy likely arises from differences in study design, statistical methods, and data integrity, emphasizing the need for robust, independent validations to confirm the role of genetic factors in AMD treatment.

#### 3.10.2. Interactions of Beta-Carotene, Smoking and Long-Term Safety

During the AREDS trial, two clinical studies highlighted the risks of beta-carotene supplementation, particularly for smokers [177]. The Beta-Carotene and Retinol Efficacy Trial, involving 18,314 individuals including smokers and those exposed to asbestos, found that daily supplementation with 30 mg of beta-carotene and 25,000 IU of vitamin A increased the risk of lung cancer [178]. After four years of follow-up, the treatment group showed a 28% higher risk of lung cancer compared to the placebo group [179].

Similarly, the Alpha-Tocopherol, Beta-Carotene Cancer Prevention Study revealed that beta-carotene supplementation (20 mg/day) increased lung cancer risk. The Beta-Carotene and Retinol Efficacy Trial also reported similar results [180]. Further analysis showed that higher risks were seen among participants who smoked more heavily or consumed more alcohol [180]. Overall, beta-carotene supplementation was not effective in preventing lung cancer and may increase its incidence in smokers, especially with higher levels of alcohol consumption [180].

#### 3.10.3. Notable Side Effects

The high doses of vitamin C, vitamin E, and zinc in the AREDS2 formula can cause concerns for some individuals. Studies have shown that daily supplementation of vitamin E at 400 IU or more may slightly increase the risk of all-cause mortality [181]. Additionally, the Selenium and Vitamin E Cancer Prevention Trial revealed a 17% higher risk of prostate cancer in men taking 400 IU of vitamin E daily over seven years [182].

High doses of vitamin C (500 mg or more) may have a pro-oxidant effect, potentially increasing the risk of cataract development. Research has suggested that vitamin C supplements may raise cataract risk by up to 38% in women aged 65 and older [183].

The AREDS2 formula contains 80 mg of zinc, double the recommended upper intake level. This elevated dose has been linked to a higher likelihood of urinary complications, as shown in a study by Aaron R. Johnson [184]. Additionally, zinc may accelerate AMD progression in individuals with specific genetic variants, such as CFH and ARMS2 [174].

### 3.11. Moving Beyond Supplements

#### 3.11.1. Dietary Recommendations: Role of a Mediterranean Diet and Other Dietary Patterns

A study by Gourgouli and colleagues on Greek patients with early or intermediate dry AMD found that adherence to the Mediterranean Diet (Med Diet) was linked to improved or stabilized AMD over a year. Those with higher adherence to the Med Diet had a 2.2 times higher likelihood of slowing progression compared to those with low adherence. Additionally, patients taking supplements had an 8.2 times higher likelihood of slowing AMD progression [185]. The Coimbra Eye Study in their case-control study reported that the study subpopulation with lower AMD prevalence reported a higher adherence to Mediterranean diet when compared to other individual food groups [186,187].

Merle and colleagues’ research from the Rotterdam and Alenior studies revealed that individuals with greater adherence to the Med Diet had a 41% lower risk of developing advanced AMD [188]. Similarly, Kim and colleagues found that increased consumption of antioxidant-rich fruits and vegetables may protect against AMD, particularly among smokers [189].

Other protective dietary factors include higher fruit intake, which reduced the likelihood of AMD, and antioxidants such as vitamins C and E, caffeine, and beta-carotene. Conversely, high-glycemic foods were associated with an increased risk of AMD [190].

#### 3.11.2. Lifestyle Changes and Their Benefits

Cigarette smoking is a well-established risk factor for AMD, and quitting smoking early is an effective strategy to reduce the risk of developing AMD [42]. Physical exercise increases energy expenditure and nutrient intake, which can improve vitamin D status, potentially lowering the risk of AMD [103]. Combining physical activity with a diet rich in plant-based foods, particularly those high in lutein, is recommended for AMD prevention.

Sedentary lifestyles contribute to inflammation and endothelial dysfunction, which are associated with AMD progression [191]. Elevated C-reactive protein levels in patients with neovascular AMD have been partly attributed to physical inactivity [192]. In contrast, regular physical activity can boost antioxidant enzyme activity, reduce oxidative stress, and improve blood pressure and lipid levels. These combined effects help lower systemic inflammation, which plays a key role in AMD development [193].

**Table 1:** Summary of the Antioxidants, their proposed mechanisms of action and effect.

Antioxidant	Study	Findings	Proposed Mechanism of Action and Effect	Reference
Beta-carotene	AREDS 1 [84] (Randomized Placebo-Controlled, Clinical Trial)	Reduced the odds of developing advanced AMD (OR—0.72, CI—0.52–0.98); Significant reduction in rates of moderate visual acuity loss (OR—0.73, CI—0.54–0.99)	Conjugated double bonds in the molecule structure help accept electrons from ROS, neutralizing free radicals and reducing oxidative stress.	Rutz, J.K. et al. [194]
Citicoline	Nashine, S. et al. [102] (in vivo study)	Downregulated pro-apoptotic genes such as BAX, Caspase-3, and Caspase-9 in AMD RPE cybrid cells and decreased reactive oxygen species (ROS) levels by 22.8%.	Aids in glutathione synthesis, decreases lipid peroxidation, ROS generation and oxidative stress	Faiq, M.A. et al. [101]
			Prevents the degeneration of RPE cells	Nashine, S. et al. [102]
Coenzyme Q10	Lee, D. et al. [98] (in vivo study on a mouse eye model)	Blocked the upregulation of NR1 and NR2A genes, and the expression of SOD2 and HO1 proteins.	Maintains mitochondrial membrane potential as an essential cofactor of the electron transport chain, supports ATP synthesis and inhibits ROS formation	Duberley, K. et al. [95]
			Ameliorates glutamate excitotoxicity and oxidative stress in the retina	Lee, D. et al. [102]
Copper	Ferns, G.A.A. et al. [89]	Reduces ROS and regulates oxidative stress	Critical cofactor in SOD enzyme (CuZnSOD)	Ferns, G.A.A. et al. [90]
Curcumin	Burugula, B. et al. [112] (in vivo study)	Significantly reduced protease-mediated retinal ganglion cell (RGC) and amacrine cell death.	Upregulates heme oxygenase 1 (HO-1) activity, decreases SOD, glutathione and other biomarkers of oxidative stress	Bucolo, C. et al. [111]
Diterpenoid Dihydrotanshinone (DHTS)	Fresta, C.G. et al. [195] (in vitro model	Reduces IL-β maturation and inflammasome activation	Antagonist at the P2X7 receptor	Fresta, C.G. et al. [195]
Lutein	AREDS2 [126] (Randomized controlled clinical trial)	18% reduction in the risk of advanced AMD progression and a 22% decrease in neovascular AMD risk	Absorbs light between 390–540 nm, and protects the retina from photochemical light damage from harmful blue light	Van Norren, D. et al. [94]
			Decreases Factor D secretion, reducing alternative complement activation, and reduces macular inflammation	Barker, F.M. et al. [95]
Mitochondrial-targeted Antioxidants (SkQ1 and MitoQ)	Skulachev, V.P. et al. [154,155] (in-vitro study on animal eye model)	Prevented early signs of retinopathy, improving retinal pigment epithelium (RPE) function and reducing lipofuscin accumulation	Specifically target Mitochondrial ROS, and the NLRP3 inflammasome, decreasing ROS and oxidative damage, preserving RPE function	Skulachev, V.P. et al. [154,155]
Resveratrol	Cosín-Tomàs, M. et al. [175] (in vivo study)	Increased the expression of genes encoding known antioxidants (catalase, copper chaperone for superoxide dismutase 1, glutathione S-transferase zeta 1)	Reduces nitric oxide levels, inhibits lipid peroxidation, and increases reduced glutathione (GSH). Neutralizes ROS and decreases oxidative damage preserving macular function	Ryan, M.J. et al. [176,177]
Saffron	Piccardi, M. et al. [172] (Longitudinal-interventional study)	Visual acuity improved by two Snellen lines compared to baseline values (0.75 to 0.9, *p* < 0.01) after oral supplementation (20 mg/day) over a period of 14 (±2) months	Modulates gene expression by regulating calcium signalling through the P2X7 receptors. Preserves RPE function and offers neuroprotection.	Corso, L. et al. [173]
Statins	Habeos, I.G. et al. [116] (in-vitro study on a rat model)	Upregulated antioxidant enzymes like HO-1 and GPX while suppressing oxidant enzymes such as NAD(P)H oxidase and myeloperoxidase	Stimulate the Nrf2/HO-1 signaling pathway, and decreases oxidative stress and ROS production.	Habeos, I.G. et al. [116]
Vitamin C	AREDS 1 [120,121] (Randomized Placebo-Controlled, Clinical Trial)	Reduced the odds of developing advanced AMD (OR—0.72, CI—0.52–0.98); Significant reduction in rates of moderate visual acuity loss (OR—0.73, CI—0.54–0.99)	Accepts electrons from ROS due to lactone ring’s hydroxyl group in the structure, neutralizes ROS and decrease oxidative stress	Englard, S. et al. [78]
Vitamin D	Lazzara et al. [109] (in vitro study on RPE cells)	Reduces MPO activity, decreases concentrations of inflammatory markers such as MMP-9, IL-1β, like TNF-alpha, iNOS, and COX-2.	Modulates inflammatory response and reduces oxidative stress.	Lazzara et al. [109] and Leal AM et al. [108]
Vitamin E	AREDS 1 [120,121] (Randomized Placebo-Controlled, Clinical Trial)	Reduced the odds of developing advanced AMD (OR–0.72, CI–0.52–0.98); Significant reduction in rates of moderate visual acuity loss (OR–0.73, CI–0.54–0.99)	Natural forms like γ-tocopherol can trap nitrogen ROS, accepts electrons from peroxyl radicals through hydrogen donation	Wong, R.S. et al. [79]
Zeaxanthin	AREDS2 [126] (Randomized controlled clinical trial)	18% reduction in the risk of advanced AMD progression and a 22% decrease in neovascular AMD risk	Xanthophyll carotenoid concentrated in the macula absorbs blue lights and protects the macula from photochemical light damage	Van Norren, D. et al. [94]
Zinc	AREDS2 [126] (Randomized controlled clinical trial)	18% reduction in the risk of advanced AMD progression and a 22% decrease in neovascular AMD risk	Induces metallothionein synthesis and stabilizes sulfhydryl groups, also acts a cofactor in CuZnSOD enzyme, neutralizes ROS and decreases oxidative stress	Swerdel, M.R. et al. [86]

AMD–age related macular degeneration, OR–Odds ratio, CI–Confidence interval, ROS–Reactive oxygen species, NR (1 and 2A)–N-methyl-D-aspartate, SOD2–superoxide dismutase-2, HO1–heme oxygenase-1, Nrf2–nuclear factor erythroid 2-related factor 2, GPX–glutathione peroxidase.

## 4. Future Perspectives

To improve the current screening work for drug development in AMD, several approaches could be considered to refine the process and enhance treatment outcomes.

One promising direction is the integration of personalized medicine. By incorporating genetic profiling, particularly variants in genes like CFH, into the screening process, we can identify patients more likely to benefit from specific treatments. This approach not only targets the underlying genetic risk factors but also helps tailor therapies based on how an individual’s genetic makeup influences disease progression and response to treatment. It could lead to more precise and effective drug development strategies.

Another avenue to explore is the use of advanced imaging techniques. Techniques like multi-modal OCT, autofluorescence, and adaptive optics can provide more detailed and early detection of retinal changes, helping to monitor the effects of treatment in real-time also using the artificial intelligence, as shown in different fields of ophthalmology [196,197,198]. These tools would also aid in identifying early biomarkers that could guide patient stratification in clinical trials, ensuring that the right candidates are selected for the most appropriate therapies.

In addition, incorporating biomarkers of oxidative stress into the screening process would be valuable. Since oxidative stress plays a central role in AMD pathogenesis, identifying individuals with higher oxidative damage could help target antioxidant therapies more effectively. This could lead to better outcomes, as treatments could be tailored to those who would benefit the most.

Longer-term longitudinal studies could also play a crucial role in improving screening processes. By assessing the impact of lifestyle factors such as diet and physical activity alongside pharmacological treatments, these studies would provide valuable insights into how lifestyle modifications interact with new therapies. This could help ensure that drug development is not only addressing the biological aspects of AMD but also considering holistic patient care.

Finally, expanding the inclusion criteria for clinical trials to encompass a broader range of disease stages and demographic groups could be beneficial. Including patients with early-stage AMD or younger individuals, for instance, may help develop treatments that are effective across a wider population, addressing the disease before it reaches advanced stages.

Incorporating these strategies into the drug screening process could significantly improve the efficiency and effectiveness of AMD therapies, providing better-targeted treatments and a more comprehensive understanding of the disease.

## 5. Conclusions

AMD is a multifaceted disease characterized by progressive vision loss primarily driven by oxidative stress and inflammation. Antioxidants have emerged as critical players in mitigating these effects, with vitamins C, E, lutein, zeaxanthin, and Coenzyme Q10 showing promise in clinical and preclinical studies. Landmark trials like AREDS and AREDS2 have underscored the benefits of specific antioxidant formulations, although their outcomes highlight the need for patient-specific approaches, particularly in light of genetic and environmental variability. Emerging therapies, such as mitochondrial-targeted antioxidants and compounds like saffron and resveratrol, are being explored for their potential roles in AMD management. However, current evidence remains preliminary, and challenges, including variable patient responses and long-term safety concerns, underscore the need for extensive clinical research before these therapies can be widely adopted. Lifestyle modifications, such as adhering to antioxidant-rich diets and increasing physical activity, while beneficial for overall health, should be viewed as complementary measures rather than standalone solutions for AMD prevention and management. A comprehensive approach combining validated therapeutic interventions with further research into novel therapies is essential to advance AMD care.

## Data Availability

Not applicable.
